# 

**Published:** 1868-02-15

**Authors:** 


*CONTENTS:*

Physiology. Investigations, etc. By Walter Hay, M.D. .......... 97

BOOK NOTICES:

Annual Abstract of Therapeutics, Materia Medica, Pharmacy and Toxicology for 1867. By A. Bouchardat .......... 110

The Half Yearly Abstract of the Medical Sciences .......... 110

Braithwaite's Retrospect .......... 110

Half Yearly Compendium of Medical Science .......... 110

EDITORIALS:

The Endoscope .......... 111

Spring Course of Instruction .......... 111

Advertising Specialties .......... 111

The Practice of Medicine by Statute .......... 112

RUSH MEDICAL COLLEGE:

Conferring of Degrees .......... 113

Address of Prof. Blaney .......... 115

Response by the Class .......... 119

Prof. Rea's Valedictory .......... 121

